# Polyunsaturated Fatty Acids, Exercise, and Cancer-Related Fatigue in Breast Cancer Survivors

**DOI:** 10.3389/fphys.2021.759280

**Published:** 2021-10-13

**Authors:** Yutaka Matsuoka, Katsunori Tsuji, Eisuke Ochi

**Affiliations:** ^1^Division of Health Care Research, Center for Public Health Sciences, National Cancer Center Japan, Tokyo, Japan; ^2^Faculty of Bioscience and Applied Chemistry, Hosei University, Tokyo, Japan

**Keywords:** omega-3 fatty acids, ω-3, cancer-related fatigue, exercise, cancer survivorship

## Abstract

Cancer-related fatigue (CRF) is one of the most frequently reported and disabling symptoms in cancer survivors. With its negative impact on the activities of daily living, work, social activities, and mood, CRF causes severe impairment of quality of life. A previous study showed that omega-6 polyunsaturated fatty acid (PUFA) supplementation unexpectedly reduced CRF compared with omega-3 PUFA supplementation and that omega-6 PUFA supplementation reduced pro-inflammatory serum markers in fatigued American breast cancer survivors. Meanwhile, a recent meta-analysis of individual patient data revealed significant benefits of exercise interventions on CRF. Recently, we completed our randomized controlled trial among early-stage Japanese breast cancer survivors, in which we examined the effect of baseline blood PUFA characteristics on change in CRF during the 12-week trial by exercise group and confirmed that increased Cancer Fatigue Scale (CFS) was associated with both docosahexaenoic acid (DHA) (*p* = 0.06) and omega-3 index (*p* = 0.08) at baseline in all participants (*n* = 46, omega-6/omega-3 ratio = 6.79, SD = 1.90). On the contrary, DHA at baseline was positively correlated with change in CRF (*r* = 0.40, *p* = 0.06) in the control group (*n* = 24, omega-6/omega-3 ratio = 7.0). Moreover, eicosapentaenoic acid (EPA) at baseline was positively correlated with leg strength (*r* = 0.39, *p* = 0.10) in the exercise group. In conclusion, blood PUFA balance might be associated with the effect of exercise on CRF. In addition, higher EPA in individuals who conducted exercise likely has a beneficial effect on muscle strength. Further investigation is needed to clarify the interaction between PUFAs and exercise for alleviating CRF.

Cancer-related fatigue (CRF) is one of the most frequently reported and disabling symptoms in cancer survivors. The primary candidates for causing fatigue are cancer treatment, particularly treatment with adjuvant chemotherapy, depression, pain, and sleep (Bower et al., 2000). With its negative impact on the activities of daily living, work, social activities, and mood, CRF causes severe impairment of quality of life. The phospholipid polyunsaturated fatty acids (PUFAs) are promising candidates for the reduction of CRF (Peppone et al., 2019) and are divided into two main types of PUFAs in the human body: the omega-6 PUFA series derived from *cis*-linoleic acid (LA, 18:2) and the omega-3 PUFA series derived from alpha-linolenic acid (ALA, 18:3) (Su, 2009). Peppone et al. (2019) demonstrated in their multicenter randomized controlled trial that omega-6 PUFA supplementation unexpectedly reduced CRF compared with omega-3 PUFA supplementation and that omega-6 PUFA supplementation reduced pro-inflammatory serum markers in fatigued American breast cancer survivors. Meanwhile, an earlier large-scale cross-sectional study found that a higher intake of omega-6 PUFAs relative to omega-3 PUFAs was associated with 1.8 times greater C-reactive protein and 2.6 times greater odds of CRF in 633 American breast cancer survivors (Alfano et al., 2012). Strikingly, the findings of these two studies are diametrically opposed, though we must keep in mind the differences in their designs. A weakness of the trial performed by Peppone et al. was that the above result was significant for only a single-item screening question but not for standard CRF measures. Furthermore, caution is needed in extrapolating the results of Americans, who consume a large amount of omega-6 PUFAs and a small amount of omega-3 PUFAs, to the population with high fish consumption.

A recent meta-analysis of individual patient data revealed significant benefits of exercise interventions on CRF (van Vulpen et al., 2020). Ochi revealed the effect of omega-3 PUFAs on improving muscle endurance, inflammatory reaction, and delayed onset muscle soreness (Ochi and Tsuchiya, 2018). We are working on clinical research to improve physical fitness and reduce CRF in breast cancer survivors through exercise, and we are also interested in analyzing whether PUFA balance in the body affects the efficacy of exercise on CRF and muscle strength.

Recently, we, in our randomized controlled trial among early-stage Japanese breast cancer survivors (Ochi et al., 2021), examined the effect of baseline blood PUFA characteristics on change in CRF assessed by the Cancer Fatigue Scale (CFS), which was designed to reflect the nature of fatigue experienced by cancer patients (Okuyama et al., 2000), during the 12 week trial by the exercise group (Table 1). Elevated CRF, defined as an increase in CFS between baseline and 12 weeks, was associated with both docosahexaenoic acid (DHA; Spearman's rank correlation *r*_s_ = 0.28, *p* = 0.06) and omega-3 index (*r*_s_ = 0.26, *p* = 0.08) at baseline in all participants (*n* = 46, omega-6/omega-3 ratio = 6.79, SD = 1.90). On the contrary, DHA at baseline was positively correlated with change in CRF (*r* = 0.40, *p* = 0.06) in the control group (*n* = 24, omega-6/omega-3 ratio = 7.0). Moreover, eicosapentaenoic acid (EPA) at baseline was positively correlated with leg strength (*r* = 0.39, *p* = 0.10) in the exercise group. In our phase II trial, the associations between PUFAs and CRF were not significant, but these trends were consistent with the findings of the trial performed by Peppone et al. We speculate that blood PUFA balance might be associated with the effect of exercise on CRF, and this effect might be clearer in cancer survivors. In addition, higher EPA in individuals who conducted exercise likely has a beneficial effect on muscle strength.

**Table 1:** Correlations between blood polyunsaturated fatty acid compositions and change in cancer-related fatigue over 12 weeks.

	**All (*****n*** **=** **46)**		**Exercise (*****n*** **=** **23)**		**Control (*****n*** **=** **23)**	
	***r*_**s**_**	***p***	***r*_**s**_**	***p***	***r*_**s**_**	***p***
**Omega-6 PUFAs**
Linoleic acid	0.06	0.70	−0.28	0.20	0.28	0.20
Arachidonic acid	0.23	0.13	0.04	0.84	0.32	0.13
Total n-6 PUFAs	0.01	0.96	−0.36	0.09	0.26	0.22
**Omega-3 PUFAs**
Eicosapentaenoic acid	0.18	0.24	0.33	0.12	0.18	0.42
Docosapentaenoic acid	0.19	0.22	0.20	0.36	0.24	0.27
Docosahexaenoic acid	0.28	0.06	0.20	0.35	0.40	0.06
Total n-3 PUFAs	0.23	0.13	0.29	0.17	0.27	0.21
Omega-3 index	0.26	0.08	0.28	0.20	0.37	0.08
Omega-6/omega-3 ratio	−0.28	0.06	−0.36	0.09	−0.28	0.19

*Cancer-related fatigue was assessed using the Cancer Fatigue Scale*.

*The exercise group underwent home-based smartphone-supported high-intensity interval training using bodyweight three times a week for 12 weeks. The control group received treatment as usual*.

*r_s_, Spearman's rank correlation coefficient*.

*PUFAs, Polyunsaturated fatty acids*.

Considering the trial performed by Peppone et al. together with ours, omega-6 PUFAs might be beneficial for reducing CRF, while omega-3 PUFAs might have no benefit regardless of fish-eating habits. As no gold-standard treatment for CRF has been established, further investigation is needed to clarify the interaction between PUFAs and exercise for alleviating CRF. Self-management such as dietary modification and exercise must be both effective and easy to be an acceptable solution in cancer survivorship care.

## Data Availability Statement

The raw data supporting the conclusions of this article will be made available by the authors, without undue reservation.

## Ethics Statement

The studies involving human participants were reviewed and approved by Institutional Review Board of the National Cancer Center Japan on 28 February 2019 (No: 2018–274). The patients/participants provided their written informed consent to participate in this study.

## Author Contributions

YM and EO conceived and designed the study. YM, KT, and EO drafted the main text and table. All authors reviewed the manuscript, contributed to the acquisition, analysis, and interpretation of data, and approved the completed version of the manuscript.

## Funding

This study was supported by the National Cancer Center Research and Development Fund (30-A-17).

## Conflict of Interest

YM has received speaker fees from Suntory, Pfizer, Mochida, Eli Lilly, Morinaga Milk, and Cimic. EO has received research support from Nippon Suisan Kaisha. The remaining author declares that the research was conducted in the absence of any commercial or financial relationships that could be construed as a potential conflict of interest.

## Publisher's Note

All claims expressed in this article are solely those of the authors and do not necessarily represent those of their affiliated organizations, or those of the publisher, the editors and the reviewers. Any product that may be evaluated in this article, or claim that may be made by its manufacturer, is not guaranteed or endorsed by the publisher.
